# Supplementary material for: Sustained-input switches for transcription factors and microRNAs are central building blocks of eukaryotic gene circuits
Source: Genome Biol. 2013 Aug 23;14(8):R85. doi: 10.1186/gb-2013-14-8-r85 (PMC4054853; doi:10.1186/gb-2013-14-8-r85)
Supplement: Additional file 5 — HTML Browsable Motif Output. Zipped folder containing all WaRSwap and FANMOD motif output, viewable in a web browser. [file gb-2013-14-8-r85-S5.ZIP › HTML_browsable_motif_output/WaRSwap_all_tair9/sigs_WSWRSLDraw-2000_all.pvals.heatmaps.html/motif_id_14_000101011_tftype_all_upstream_-3000_0.html]

```
BG_MODEL = Rnew2
MOTIF_ID = 14_000101011
TF_TYPE = all
UPSTREAM = -3000_0


PVals
FN_0.2	FN_0.4	FN_0.6	FN_0.8
dg_60.genes	0.387	0.011	0.008	0.765
dg_70.genes	0.308	0.007	0.004	0.736
dg_80.genes	0.772	0.148	0.095	0.611

ZScores
FN_0.2	FN_0.4	FN_0.6	FN_0.8
dg_60.genes	0.246	2.741	3.195	-0.916
dg_70.genes	0.494	2.998	3.35	-0.86
dg_80.genes	-0.843	1.081	1.246	-0.737

StDevs
FN_0.2	FN_0.4	FN_0.6	FN_0.8
dg_60.genes	185.536	182.885	92.775	21.015
dg_70.genes	180.197	177.794	90.448	21.155
dg_80.genes	145.01	144.558	82.258	16.542
```
